# Estimation of Vertical Leaf Nitrogen Distribution Within a Rice Canopy Based on Hyperspectral Data

**DOI:** 10.3389/fpls.2019.01802

**Published:** 2020-02-13

**Authors:** Jiaoyang He, Xiangbin Zhang, Wanting Guo, Yuanyuan Pan, Xia Yao, Tao Cheng, Yan Zhu, Weixing Cao, Yongchao Tian

**Affiliations:** ^1^National Engineering and Technology Center for Information Agriculture, Nanjing Agricultural University, Nanjing, China; ^2^Key Laboratory for Crop System Analysis and Decision Making, Ministry of Agriculture, Nanjing, China; ^3^Jiangsu Key Laboratory for Information Agriculture, Nanjing Agricultural University, Nanjing, China; ^4^Jiangsu Collaborative Innovation Center for Modern Crop Production, Nanjing Agricultural University, Nanjing, China

**Keywords:** rice, leaf nitrogen concentration, vegetation index, vertical distribution, remote sensing

## Abstract

Accurate estimations of the vertical leaf nitrogen (N) distribution within a rice canopy is helpful for understanding the nutrient supply and demand of various functional leaf layers of rice and for improving the predictions of rice productivity. A two-year field experiment using different rice varieties, N rates, and planting densities was performed to investigate the vertical distribution of the leaf nitrogen concentration (LNC, %) within the rice canopy, the relationship between the LNC in different leaf layers (LNC_Li_, i = 1, 2, 3, 4), and the relationship between the LNC_Li_ and the LNC at the canopy level (LNC_Canopy_). A vertical distribution model of the LNC was constructed based on the relative canopy height. Furthermore, the relationship between different vegetation indices (VIs) and the LNC_Canopy_, the LNC_Li_, and the LNC vertical distribution model parameters were studied. We also compared the following three methods for estimating the LNC in different leaf layers in rice canopy: (1) estimating the LNC_Canopy_ by VIs and then estimating the LNC_Li_ based on the relationship between the LNC_Li_ and LNC_Canopy_; (2) estimating the LNC in any leaf layer of the rice canopy by VIs, inputting the result into the LNC vertical distribution model to obtain the parameters of the model, and then estimating the LNC_Li_ using the LNC vertical distribution model; (3) estimating the model parameters by using VIs directly and then estimating the LNC_Li_ by the LNC vertical distribution model. The results showed that the LNC in the bottom of rice canopy was more susceptible to different N rates, and changes in the LNC with the relative canopy height could be simulated by an exponential model. Vegetation indices could estimate the LNC at the top of rice canopy. R_705_/(R_717_+R_491_) (R^2^ = 0.763) and the renormalized difference vegetation index (RDVI) (1340, 730) (R^2^ = 0.747) were able to estimate the parameter “a” of the LNC vertical distribution model in indica rice and japonica rice, respectively. In addition, method (2) was the best choice for estimating the LNC_Li_ (R^2^ = 0.768, 0.700, 0.623, and 0.549 for LNC_L1_, LNC_L2_, LNC_L3_, and LNC_L4_, respectively). These results provide technical support for the rapid, accurate, and non-destructive identification of the vertical distribution of nitrogen in rice canopies.

## Introduction

Nitrogen (N) is a key nutrient resource required for vegetation growth (Aerts and Chapin, 1999; Wang et al., 2005; Xu et al., 2012), which affects the development of important photosynthetic organs (leaves, etc.), enzymes (RuBisCO, etc.), and pigments (chlorophyll, etc.) (Gu et al., 2014). Therefore, the timely acquisition of N accumulation and distribution data within the vegetation canopy can provide insights for vegetation growth and help improve the prediction accuracy of vegetation productivity (Hikosaka et al., 2016; Gu et al., 2017).

During the growth and development of vegetation, the canopy structure changes continuously, which leads to differences in light reception at the vertical level of the canopy. Due to the short phenological cycles of certain vegetation (such as rice and wheat), it is unprofitable for the plants to invest in slow structural changes to adapt to changes in the light environment. As a transferable nutrient element (Wang et al., 2005), N is easy to transport in plants and represents an effective method of maximizing photosynthesis by N reallocation (Dreccer et al., 2000; Bertheloot et al., 2012; D'Odorico et al., 2019). Hirose and Werger (1987) explained the mechanism (the optimization theory) of non-uniform vertical N distribution from the aspect of light distribution and indicated that leaves under better light conditions can obtain more N than those under poorer light conditions and thus can maximize the total photosynthesis in the canopy. Based on these results, Chen et al. (1993) proposed the coordination theory, which advocates that there is a potential trade-off in the investment in structures that increase the capacity for light capture and carbon assimilation because of the huge N consumption by the above two processes.

In recent years, the non-uniform vertical N distribution has been reported for various plant canopies (Archontoulis et al., 2011; Li et al., 2015; Hikosaka et al., 2016; Zhao et al., 2017; Gara et al., 2018; Ye et al., 2018), and the influencing factors could be roughly divided into two categories: environmental factors and vegetation factors. In terms of environmental factors, the light condition is one of the key factors that causes the non-uniform distribution of N. A number of studies have shown that the specific leaf nitrogen (SLN) in different leaf layers is closely related to the light conditions (Dreccer et al., 2000; Archontoulis et al., 2011; Hikosaka, 2014; Coble and Cavaleri, 2015). Different N rates also affect the vertical distribution; results from the study by Hikosaka et al. (1994) showed that the N gradient is steeper under low N treatments in crop canopies. Dreccer et al. (2000) showed that canopy expansion is limited under low N rates, and the retransport of N affects the vertical distribution of SLN. Studies have also shown that air temperature is negatively correlated with the coefficient of foliage nitrogen allocation (kn, Hirose and Werger, 1987) and affects the distribution of photosynthetic N (Kellomäki and Wang, 1997; Kattge and Knorr, 2007). In terms of vegetation factors, the differences in canopy structure (such as the leaf area index and leaf angle) that cause changes in light conditions are the main reasons for the non-uniform distribution of N (Archontoulis et al., 2011; Hikosaka et al., 2016; Ye et al., 2018). Moreover, differences in growth periods (or differences in leaf age) also affect the distribution of N within the canopy (Dreccer et al., 2000; Gastal and Lemaire, 2002; Winterhalter et al., 2012; Chen et al., 2014; Ye et al., 2018).

As a rapid and non-destructive method, remote sensing has been widely used to determine the vegetation N status (Mitchell et al., 2012; Oerke et al., 2016; Song et al., 2016; Chemura et al., 2018; Prey and Schmidhalter, 2019); however, such analyses assume that the vegetation canopy is uniform and seldom consider the N distribution. Vertical heterogeneity of the canopy is being increasingly recognized in remote estimates of vegetative properties (Jia et al., 2013; Li et al., 2013; Liu et al., 2015; Li et al., 2016; Zhao et al., 2017). Ciganda et al. (2012) reported that the red edge chlorophyll index (CI_red-edge_) could effectively monitor the chlorophyll content of the top 7 to 9 leaves in the maize canopy. Ye et al. (2018) developed a simple ratio vegetation index—SR (736, 812) to monitor the leaf N density (LND, g N m^-2^ soil) of the upper, middle, and lower leaves in the upright leaf maize canopy. Winterhalter et al. (2012) proposed the R780/R740, and it was positively and curvilinearly related to the N uptake profile of the maize foliage and capable of detecting the N uptake of each leaf layer, even at the lowest layers. Li et al. (2018) identified several bands as effective wavelengths for assessing the vertical LNC distribution in different leaf layers. Luo et al. (2016) investigated the vertical distribution characteristics of the N concentration within the reed canopy and developed a model that considered the vertical distribution patterns of the N concentration and that in the effective canopy layers to estimate the total N concentration of the whole reed canopy based on the PPR/NDVI.

The above studies explored the possibility of estimating the vertical distribution of vegetative properties and analyzed the detection depth of VIs in different vegetation canopies. However, few studies have combined the vertical distribution characteristics of vegetative properties with remote sensing methods. Our specific aims are to (1) explore the temporal and spatial distribution characteristics of the LNC in a rice canopy at different growth stages; (2) compare the correlation between different VIs and LNC in different leaf layers of the rice canopy; and (3) develop an effective method of estimating the LNC of each leaf layer within the rice canopy.

## Materials and Methods

### Study Area and Experimental Details

Two rice field experiments with different N rates, planting densities, and rice cultivars were conducted at the experimental station of the National Engineering and Technology Center for Information Agriculture in 2015 and 2016 (**Supplementary Figure 1**). The experimental station is located in Rugao City, Jiangsu Province, China (120°45′E, 32°16′N). A *Japonica* rice cultivar (Wuyunjing24) with erect leaf and an *Indica* rice cultivar (Eryou728) with spread leaf were involved in the experiments. Two N rates (N1 = 100 kg N ha^-1^ and N3 = 300 kg N ha^-1^) were applied with three planting densities (D2 = 22.22 plants m^-2^, D3 = 16.66 plants m^-2^, and D4 = 13.33 plants m^-2^). Each treatment in the two experiments had three replicates. The plot area was 30 m^2^, with dimensions 6 × 5 m. Two experiments were seeded on May 15; one was transplanted into the paddy field on June 15 in 2015, and the other one on the same date in 2016. The experimental details are shown in **Supplementary Table 1**, and He et al. (2019) provided more information on the field manage measures.

### Spectral Measurements

A spectrometer (FieldSpec4 Standard-Res, Analytical Spectral Device, Boulder, CO, USA) with a spectral range of 350–2500 nm and view angle of 25° was used to take canopy reflectance measurements. In each plot, 5 fixed points were selected for spectral measurements, and the replicate reflectance spectra were averaged to represent the sample spectrum. All reflectance spectra were taken at 1.0 m above the rice canopy between 10:00 and 14:00 under cloud-free conditions. Previous VIs for LNC estimation are summarized in **Supplementary Table 2**.

### Field Data Collection

Repeated destructive sampling was carried out after each canopy spectral reflectance measurement. Before destructive sampling, 10 plants from each experimental plot were randomly selected to determine the tiller number and canopy height (H, cm), and the average were used as the tiller number and canopy height of each plot. The basic statistics of the canopy height are shown in **Supplementary Table 3**. Three plants (for which the tiller number is the same or similar to the tiller number of the plot) from each plot were selected and brought back to the laboratory, and they were clipped into 3 equal layers at the pre-heading stage (58 DAT to 86 DAT) and into 4 equal layers at the post-heading stage (after 86 DAT) after removing their roots. Each leaf layer (Li, i = 1, 2, 3, 4) and each accumulative leaf layer (ALi, i = 1, 2, 3, 4) are shown in **Supplementary Figure 2**. The relative canopy height (Hr) of each leaf layer from top to bottom were 0.840, 0.500, and 0.160 at the pre-heading stage and 0.875, 0.625, 0.375, and 0.125 at the post-heading stage.

For each layer, the samples were divided into leaves and stems (for top layer, including panicles after heading stage), and leaves were further separated into the green and yellow parts. The leaf area of green leaves was measured using a leaf area meter (LI-3100C, LI-COR Inc., NE, USA). The dry weight (DW, g) of the samples were obtained after oven drying at 80°C to a constant weight. The leaf area index (LAI, m^2^ leaf m^-2^ soil) and leaf dry weight (LDW, g leaf m^-2^ soil) were calculated in each plot from the planting densities. The LNC (%) was measured using the C-N vario MACRO cube (Elementar, Hanau, Germany). The leaf N concentration ratio (LNC_R_) and the agronomic parameters of each accumulative leaf layer were calculated as follows:

(1) $$LNC_{Ri}=\frac{LNC_{Li}\times DW_{Li}}{LNC_{Canopy}\times DW_{Canopy}}$$

(2) $$DW_{ALi}=\sum_{1}^{i}DW_{Li}$$

(3) $$LNC_{ALi}=\frac{\sum_{1}^{i}\left(LNC_{Li}\times DW_{Li}\right)}{DW_{ALi}}$$

where *i* is the layer with values of *i* = 1, 2, 3, 4 from the top to bottom of the rice canopy; *DW_Li_*, *LNC_Li_*, and *LNC_Ri_* are the DW, LNC, and LNC_R_ of Li, respectively; and *DW_ALi_* and *LNC_ALi_* are the DW and LNC of ALi, respectively, with *LNC_ALi_* = LNC_Canopy_ when i is the lowest layer (i = 3 in pre-heading stages and i = 4 in post-heading stages).

### LNC Vertical Distribution Model

Many quantitative models for the vertical leaf nitrogen distribution within plant canopies have been proposed in previous studies (Hirose et al., 1988; Lemaire et al., 1991; Anten et al., 1998). The model [Eqn (4)] proposed by Lemaire et al. (1991) has only one variable, i.e., the depth from the top of the canopy, and it is easy to apply to remote sensing. In this study, we improved this model as an LNC distribution model [Eqn (5)] with an independent variable, i.e., relative canopy height, to make it suitable for rice at different growth stages.

(4) $$N_{L}=aexp\left(-bZ_{D}\right)$$

(5) $$LNC_{Li}=aexp\left(bH_{ri}\right)$$

where *N_L_* is the N content per unit leaf area within a canopy; *Z_D_* is the depth from the top of canopy; *LNC_Li_* is the LNC of Li; *Hri* is the relative canopy height of Li; and *a* and *b* are the regression parameters.

### Different Methods for Estimating LNC_Li_

**Figure 1:**
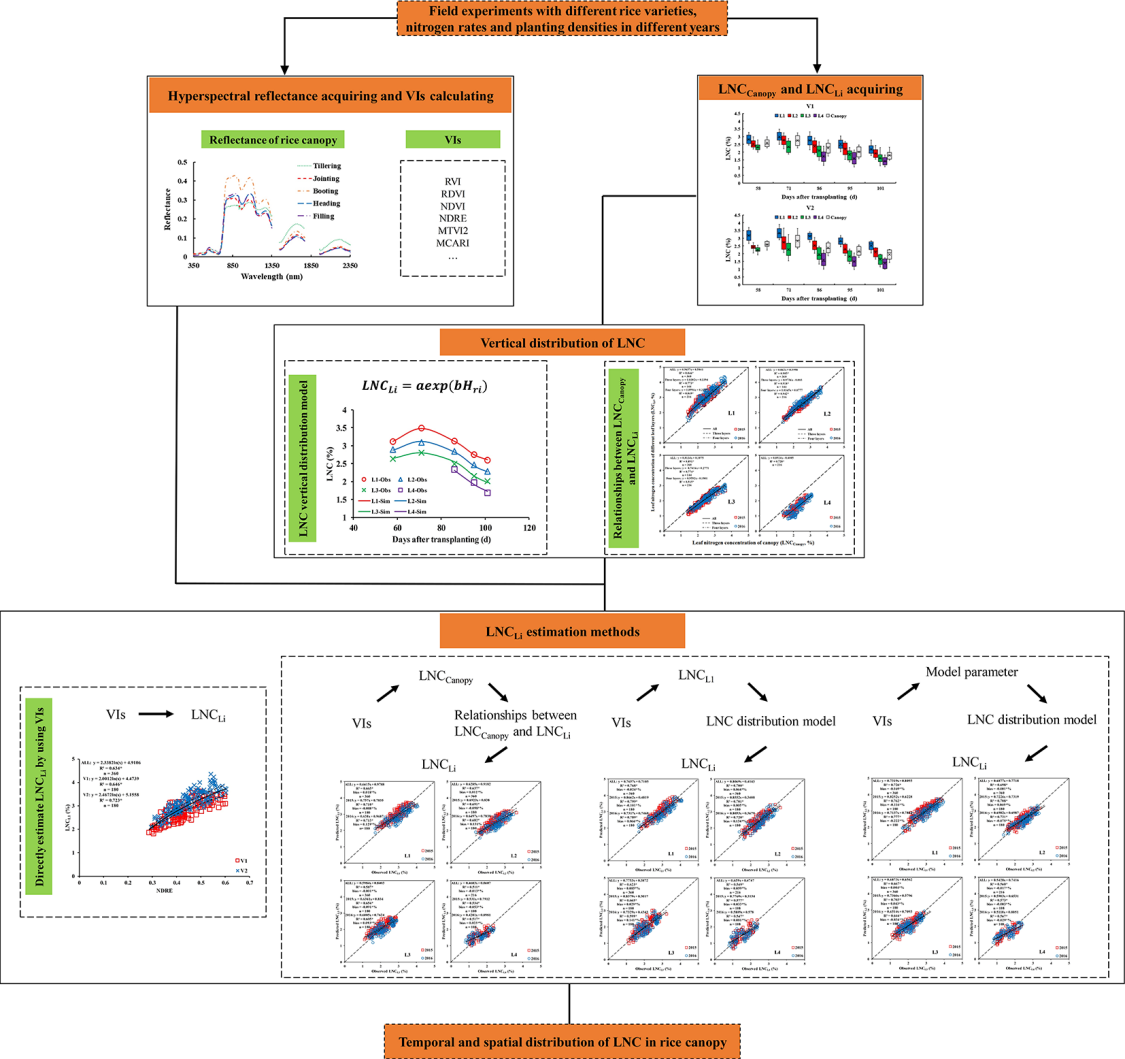
Flowchat for establishing the optimal LNC_Li_ estimation model.

Three methods were used to estimate the LNC_Li_: (1) estimating the LNC_Canopy_ by VIs and then estimating the LNC_Li_ based on the relationship between the LNC_Li_ and LNC_Canopy_; (2) estimating the LNC in any leaf layer of the rice canopy by VIs, inputting the results into the LNC vertical distribution model [Eqn (5)] to obtain the model parameters, and then estimating the LNC_Li_ by the LNC vertical distribution model; and (3) estimating the model parameters by using VIs directly and then estimating the LNC_Li_ by the LNC vertical distribution model. **Figure 1** shows the main steps for the whole procedure.

## Results

### Temporal and Spatial Distribution Characteristics of the LNC in the Rice Canopy

**Figure 2:**
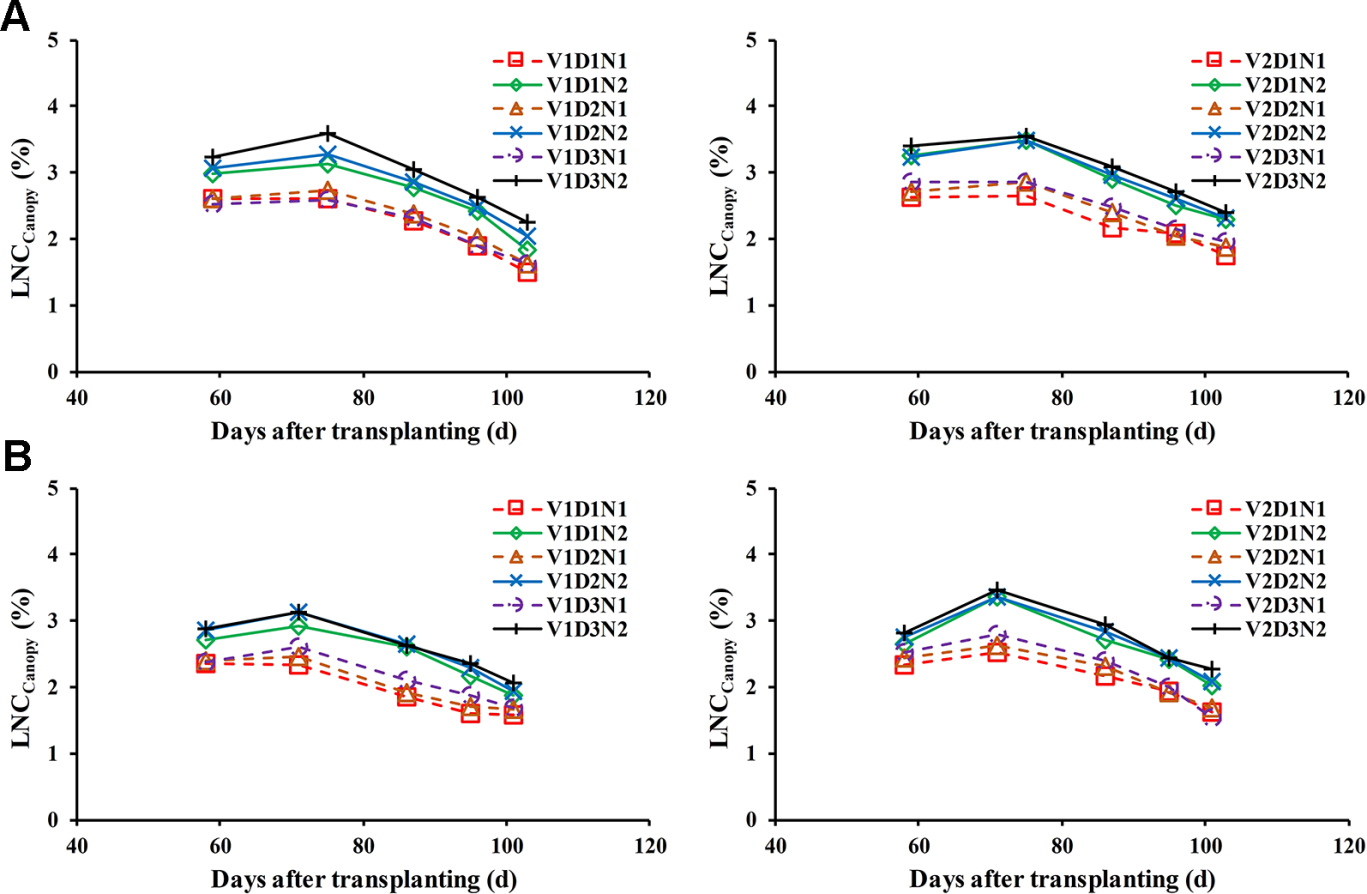
Changes of the LNC_Canopy_ during the whole growth stages under different treatment in 2015 **(A)** and 2016 **(B)**. Jointing fertilizer was applied at approximately 60 days after transplanting (DAT).

Changes of the LNC_Canopy_ during whole growth stages in different treatments and years are shown in **Figure 2**. The results showed that the LNC_Canopy_ decrease with increases in the days after transplanting in all treatments when the effect of nitrogen fertilizer application was neglected, which caused the LNC_Canopy_ to increase slightly.

**Figure 3:**
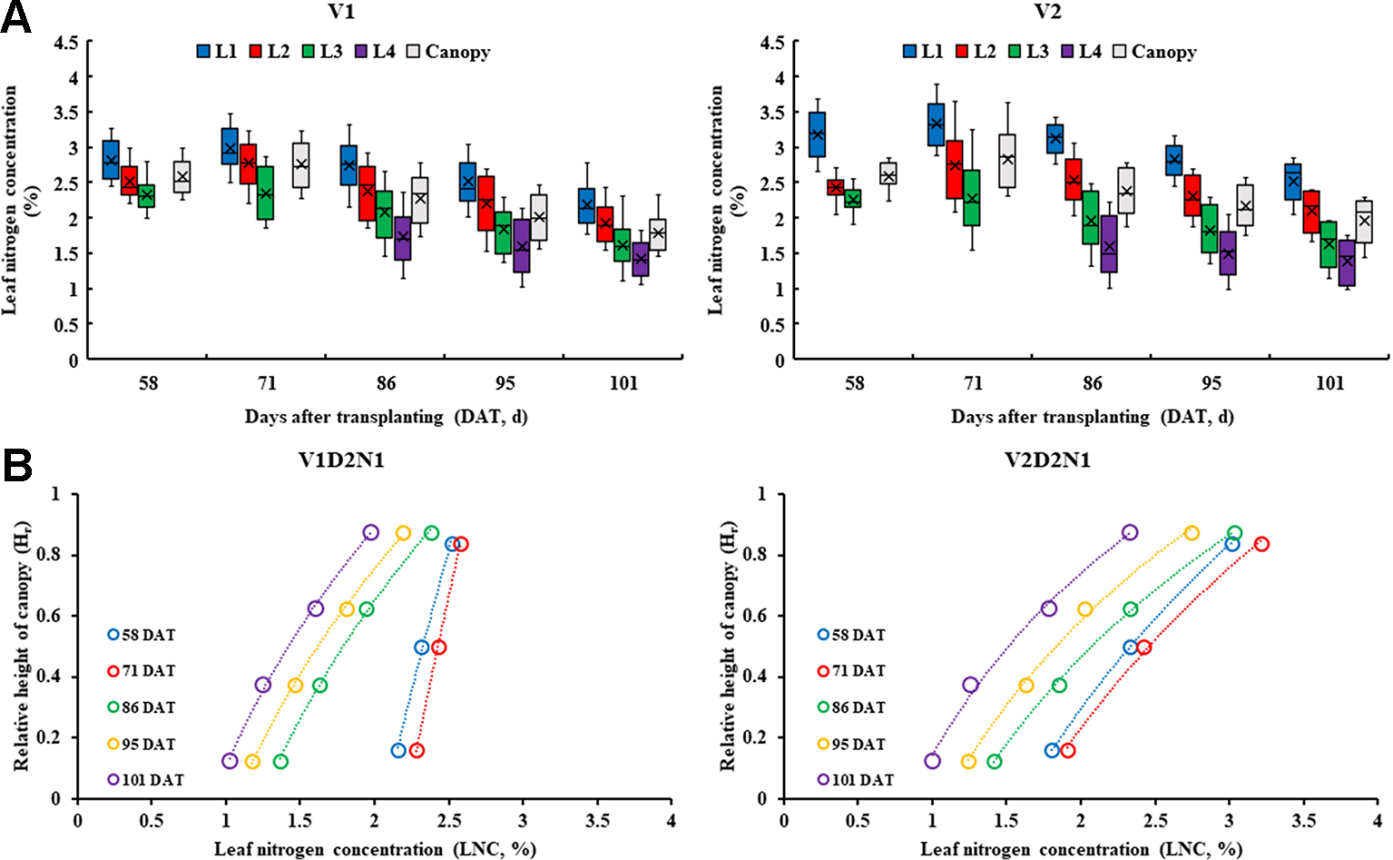
Data distribution characteristics of the LNC_Li_ and LNC_Canopy_ at different growth period in 2015 **(A)** and the vertical distribution characteristics of the LNC in rice canopy, take V1D1N1 and V2D1N1 for example **(B)**.

The data distribution characteristics of the LNC_Li_ and LNC_Canopy_ at different growth periods (**Figure 3A**) indicated that the values of the LNC_L2_ and LNC_Canopy_ are similar in all treatments during whole growth stages. Compared with V1, there were significant differences between the LNC_L1_ and LNC_L4_ in V2. The LNC presented a decreasing trend from top to bottom within the rice canopy, and the LNC of each leaf layer decreased along with the days after transplanting (**Figure 3B**).

**Figure 4:**
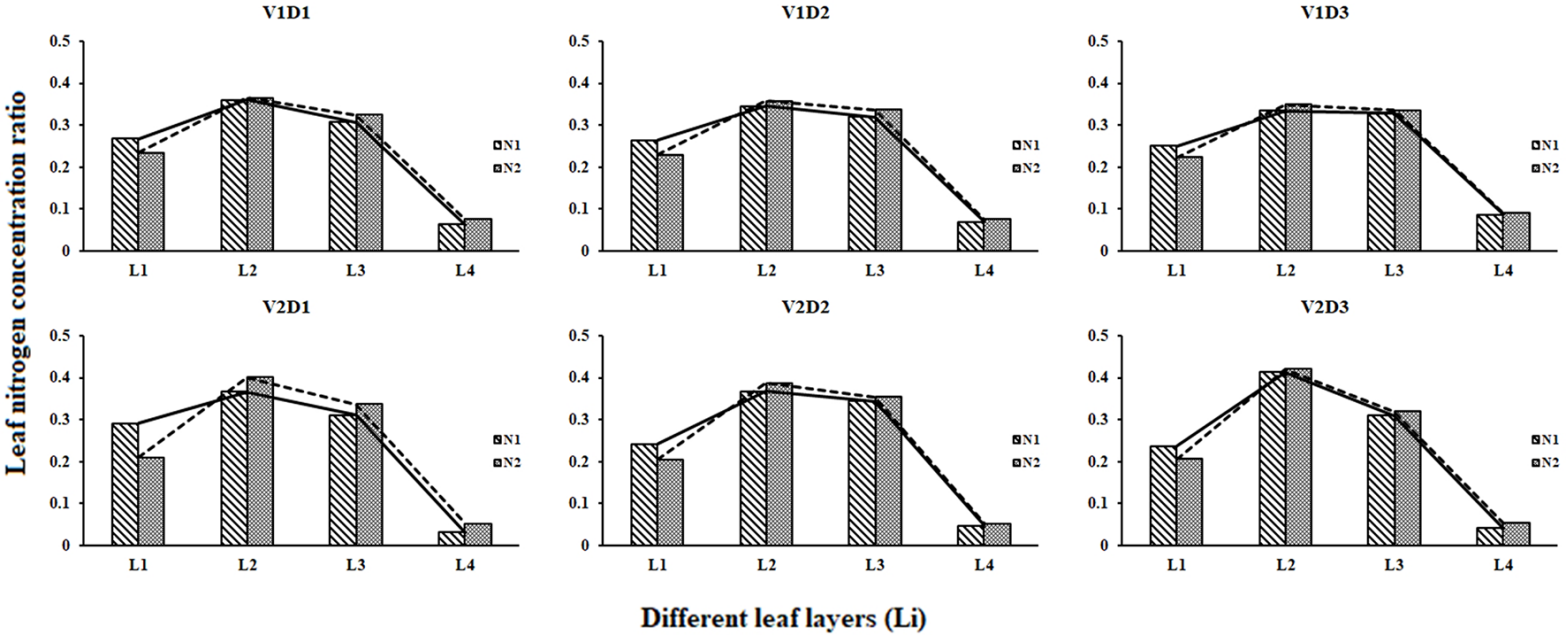
Leaf nitrogen concentration ratio of different leaf layers under different treatments (2015).

The leaf N concentration ratio of each leaf layer changed little in different treatments during whole growth stages (**Figure 4**). The LNC_R2_ was the highest (0.34 – 0.37 for V1, and 0.38 – 0.43 for V2), and the LNC_R4_ was the lowest (0.06 – 0.09 for V1, and 0.03 – 0.05 for V2). The differences among the LNC_R_ between high N rates and low N rates decreased as the planting densities decreased.

### Relationships of LNC_Canopy_ With LNC_Li_ and LNC_ALi_

**Figure 5:**
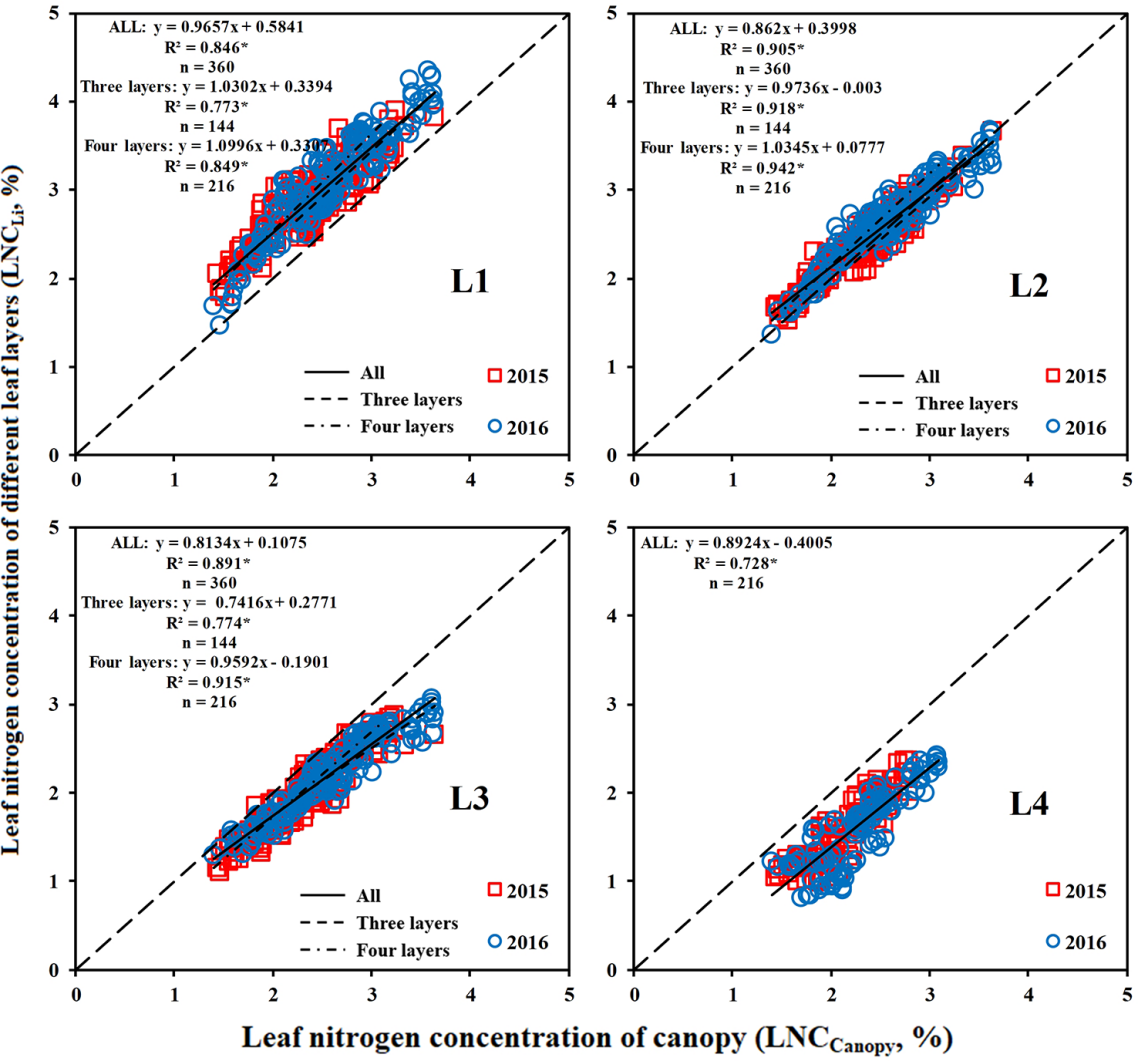
Relationship between the LNC_Li_ and LNC_Canopy_. * means accurate to 0.001.

The LNC of the middle and upper leaf layers had a closer relationship with the LNC_Canopy_ than that of the lower leaf layer (**Figure 5**). The LNC_L1_ (upper leaf layer) was significantly higher than the LNC_Canopy_, whereas the LNC_L3_ and LNC_L4_ (lower leaf layer) were lower than the LNC_Canopy_. Overall, the relationship between the LNC_Li_ and LNC_Canopy_ was stable over the years.

**Figure 6:**
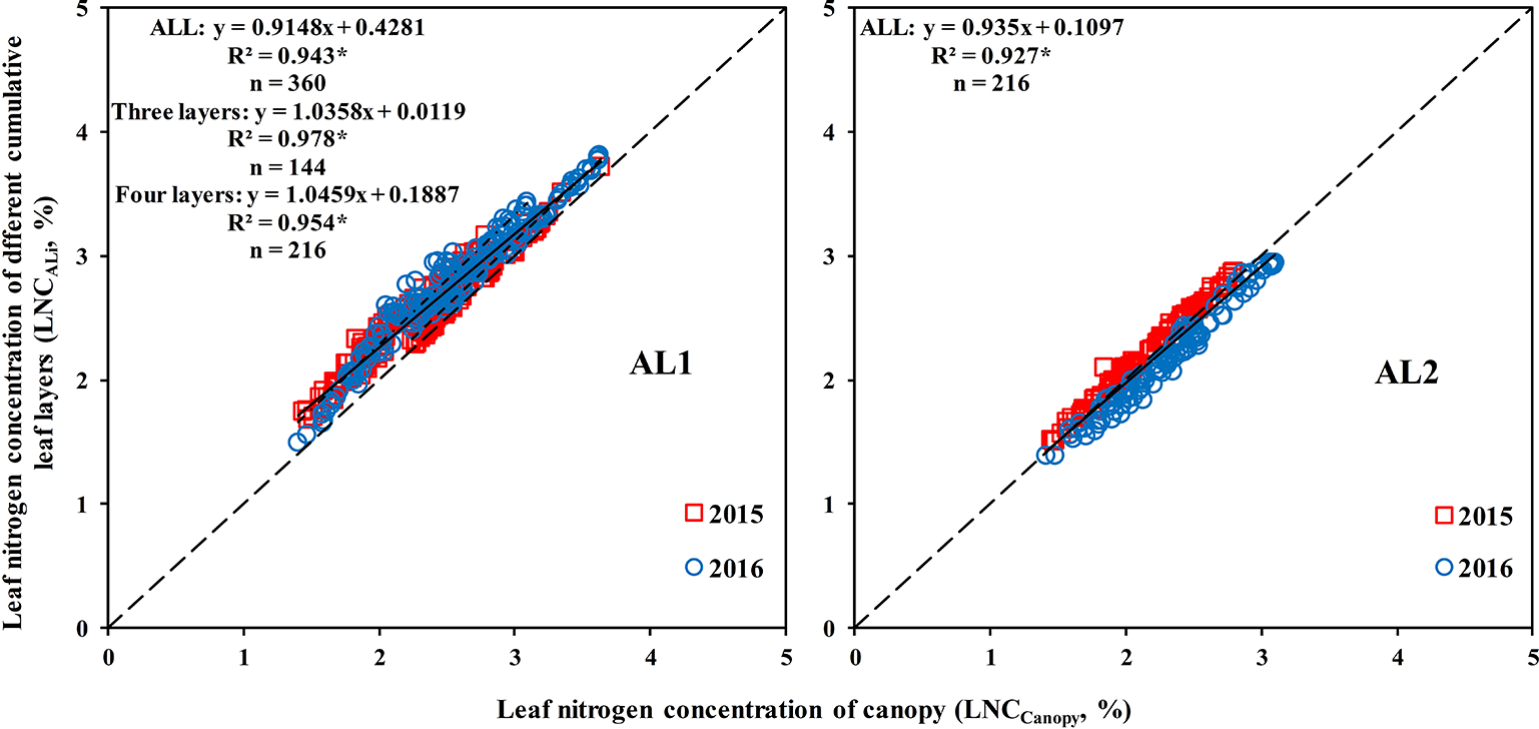
Relationship between the LNC_ALi_ and LNC_Canopy_. * means accurate to 0.001.

A good correlation was observed between the LNC_ALi_ and LNC_Canopy_, and the coefficient of determination (R^2^) could reach more than 0.9 (**Figure 6**). The LNC_AL2_ could best represent the LNC_Canopy_ when the canopy was divided into three leaf layers, which indicated that from top to bottom, 67% of the canopy can represent the whole canopy. The LNC_AL3_ could represent the LNC_Canopy_ best when the canopy was divided into four leaf layers, which demonstrated that from top to bottom, 75% of the canopy can represent the whole canopy.

### Effects of Different N Rates and Planting Densities on the LNC_Li_

**Figure 7:**
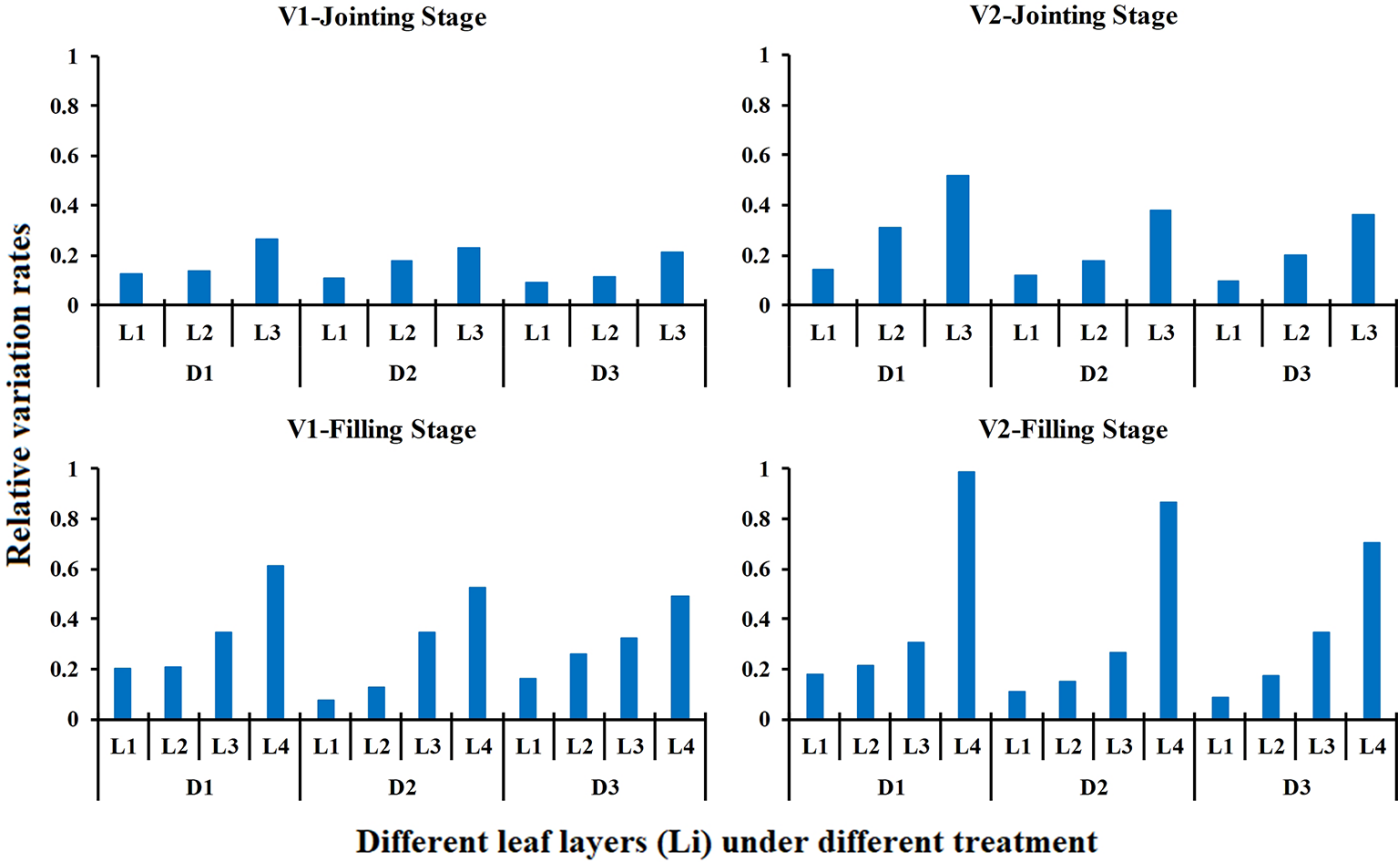
Relative variation rate of the LNC_Li_ in response to different N rates in different growth stage (2015). The relative variation rate is the product of the nitrogen traits under high nitrogen treatment minus the nitrogen traits under low nitrogen treatment over the nitrogen traits under low nitrogen treatment.

The effects of different N rates and planting densities on the LNC_Li_ in different growth stages are shown in **Figure 7**. The results indicated that the effect of different N rates on the LNC of each leaf layer increased from the top to the bottom of the canopy, and the bottom leaf layer (L3) was most affected by the N application level at the jointing stage. Similar rules were found during the filling stage, and the impact increased slightly. The bottom leaf layer (L4) was still the leaf layer that was most affected by the N rate. The effects of different N rates on the LNC_Li_ did not differ between the two rice varieties. Compared with N rate, planting densities has less effect on LNC_Li_. For the same variety and the same growth period, the relative variation rates of LNC in middle and upper leaf layers remained stable under varied planting densities. However, the relative variation rates of LNC in the bottom leaf layer decreased gradually with decreased planting densities.

### LNC Vertical Distribution Model Verification and Parameter Acquisition

**Figure 8:**
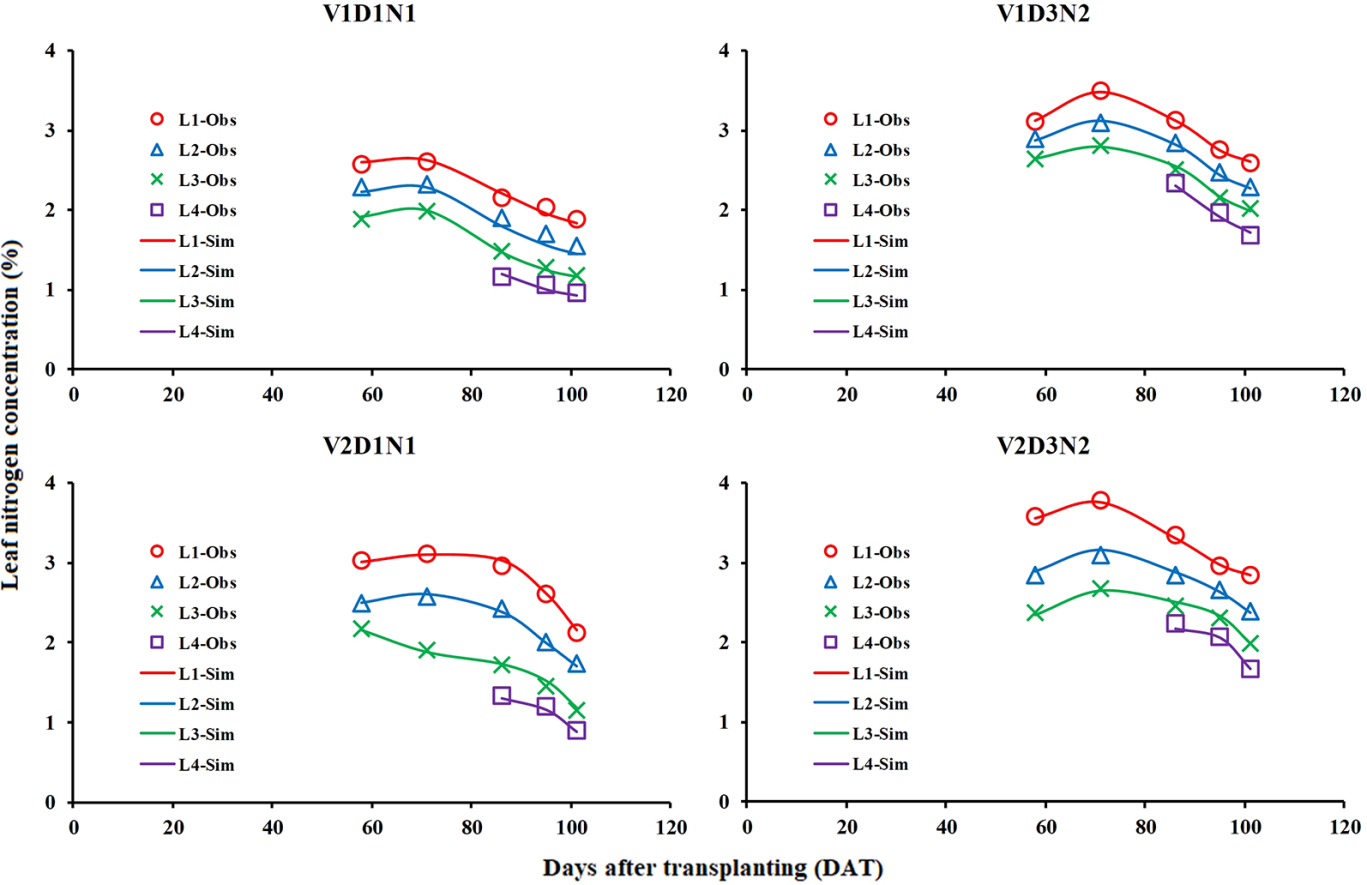
Comparison of simulated (Sim) and observed (Obs) distribution of leaf nitrogen concentration in rice canopy (take V1D1N1, V1D3N2, V2D1N1, and V2D3N2 for example).

The measured LNC_Li_ values in two field experiments were used to test the LNC vertical distribution model (**Figure 8**) and obtain the model parameters (**Table 1**). The results showed that the LNC_Li_ could be better simulated in different varieties, treatments, and growth stages by using the LNC vertical distribution model proposed in this study (average R^2^ is higher than 0.97).

**Table 1:** Basic statistics of the parameters of the vertical distribution of the leaf nitrogen content model.

		V1				V2			
		Mean	SD	Min.	Max.	Mean	SD	Min.	Max.
**Three layers**	**a**	2.2381	0.3485	1.6980	2.9680	2.0415	0.3893	1.3420	2.7050
	**b**	0.3947	0.1017	0.2074	0.6435	0.6475	0.1874	0.2652	1.0580
	**R^2^**	0.9787	0.0201	0.9093	1.0000	0.9826	0.0194	0.9147	1.0000
	**RMSE**	0.0657	0.0406	0.0001	0.1550	0.0914	0.0688	0.0007	0.2890
**Four layers**	**a**	1.5368	0.3771	0.8923	2.3370	1.3746	0.3905	0.7599	2.2010
	**b**	0.6709	0.1472	0.3154	0.9497	0.9360	0.2340	0.4877	1.3870
	**R^2^**	0.9713	0.0235	0.9008	0.9991	0.9831	0.0143	0.9299	1.0000
	**RMSE**	0.0788	0.0405	0.0117	0.2201	0.0941	0.0492	0.0027	0.2487

The range of values of model parameters “a” and “b” was slightly different between the rice varieties and different growth stages. In the same period, the parameter “a” of V1 was higher than that of V2, with the value ranging between 1.6980 and 2.9680; however, the parameter “b” of V1 was lower than that of V2, with the value ranging between 0.2074 and 0.6435. The value of parameter “a” decreased as the growth period advanced; however, the value of parameter “b” increased. Parameter “a” was well correlated with parameter “b” in the two rice varieties (R^2^ values were 0.84 and 0.87, respectively) and decreased as parameter “b” increased.

### Relationships of the VIs With LNC_Li_ and Parameter “a”

The relationship between the LNC_Li_ and VIs was tested (**Tables 2**, **3**, **4**), and the results showed that the VIs could estimate the LNC_Canopy_ and were well correlated with the LNC_Li_. The estimation accuracy of the VIs for the LNC_Canopy_, LNC_L1_, LNC_L2_, LNC_L3_, and LNC_L4_ decreased in turn, and the corresponding optimal VIs were the NDDA (R^2^^=^ 0.684), NDRE (R^2^^=^ 0.631), NDRE (R^2^^=^ 0.589), RVI (R^2^^=^ 0.516), and RVI (R^2^^=^ 0.507) when combined for the two rice varieties. The relationship between VIs and LNC_Li_ did not change significantly between years (**Table 3**); the estimation accuracy of a single year was better than those of two years. In addition, the optimal VIs for estimating LNC of each leaf layer was basically unchanged. Different VIs showed varied change modes in different planting densities (**Table 4**). At the high planting density, VIs showed the highest accuracy in estimating LNC_Canopy_ and LNC_Li_. However, the estimation accuracy of some VIs (e.g. RDVI, NDRE, and MTVI2) decreased gradually, while other VIs (e.g. RVI, NDVI and CI) decreased first and then increased at medium and low planting densities.

**Table 2:** Coefficients of determination (R^2^) for the relationship between the vegetation indices and the leaf nitrogen concentration in different rice cultivars.

	LNC_Canopy_			LNC_L1_			LNC_L2_			LNC_L3_			LNC_L4_		
	V1	V2	V1+V2	V1	V2	V1+V2	V1	V2	V1+V2	V1	V2	V1+V2	V1	V2	V1+V2
**RVI**	0.61	0.73	0.67	0.58	0.70	0.59	0.57	0.56	0.56	0.55	0.54	0.52	0.65	0.50	0.51
**NDVI**	0.43	0.69	0.54	0.56	0.72	0.65	0.55	0.57	0.57	0.30	0.51	0.33	0.32	0.40	0.25
**CI**	0.66	0.72	0.68	0.64	0.70	0.62	0.62	0.53	0.58	0.53	0.52	0.49	0.62	0.49	0.48
**OSAVI**	0.13	0.17	0.15	0.16	0.18	0.27	0.16	0.18	0.16	0.07	0.23	0.07	0.06	0.36	0.05
**NDRE**	0.65	0.73	0.68	0.65	0.72	0.63	0.63	0.56	0.59	0.54	0.53	0.50	0.63	0.48	0.45
**SIPI**	0.18	0.61	0.35	0.31	0.66	0.48	0.31	0.54	0.43	0.13	0.44	0.20	0.21	0.32	0.18
**MTVI2**	0.11	0.15	0.13	0.12	0.17	0.25	0.13	0.17	0.40	0.05	0.22	0.05	0.03	0.33	0.03
**MCARI**	0.49	0.43	0.33	0.36	0.38	0.15	0.39	0.23	0.22	0.51	0.25	0.32	0.40	0.02	0.17
**TCARI**	0.39	0.48	0.36	0.42	0.49	0.22	0.42	0.33	0.28	0.45	0.28	0.32	0.35	0.04	0.14
**NDDA**	0.67	0.71	0.68	0.63	0.69	0.60	0.62	0.53	0.57	0.55	0.52	0.51	0.66	0.49	0.50
**R_705_/(R_717_+R_491_)**	0.70	0.65	0.66	0.63	0.59	0.58	0.63	0.42	0.52	0.56	0.45	0.47	0.42	0.47	0.30
**TBDR**	0.57	0.70	0.62	0.55	0.64	0.53	0.55	0.50	0.52	0.52	0.51	0.48	0.56	0.50	0.43
**MCARI/MTVI2**	0.66	0.60	0.58	0.57	0.56	0.41	0.56	0.37	0.41	0.66	0.41	0.52	0.59	0.26	0.41
**TCARI/OSAVI**	0.52	0.59	0.50	0.51	0.59	0.36	0.50	0.40	0.38	0.58	0.36	0.45	0.40	0.12	0.25

The results were accurate to 0.01.

**Table 3:** Coefficients of determination (R^2^) for the relationship between the vegetation indices and the leaf nitrogen concentration in different years.

	LNC_Canopy_		LNC_L1_		LNC_L2_		LNC_L3_		LNC_L4_	
	2015	2016	2015	2016	2015	2016	2015	2016	2015	2016
**RVI**	0.74	0.70	0.81	0.61	0.68	0.58	0.67	0.54	0.61	0.56
**NDVI**	0.47	0.60	0.59	0.56	0.55	0.56	0.38	0.30	0.37	0.27
**CI**	0.72	0.74	0.81	0.69	0.64	0.62	0.53	0.54	0.52	0.46
**OSAVI**	0.32	0.25	0.56	0.51	0.33	0.26	0.16	0.01	0.16	0.01
**NDRE**	0.72	0.72	0.82	0.66	0.66	0.61	0.54	0.53	0.54	0.44
**SIPI**	0.27	0.32	0.35	0.24	0.41	0.38	0.18	0.12	0.24	0.09
**MTVI2**	0.27	0.25	0.12	0.13	0.28	0.16	0.13	0.01	0.11	0.00
**MCARI**	0.34	0.35	0.53	0.37	0.23	0.28	0.22	0.21	0.12	0.20
**TCARI**	0.31	0.40	0.55	0.37	0.25	0.30	0.25	0.24	0.14	0.15
**NDDA**	0.74	0.74	0.82	0.68	0.65	0.62	0.57	0.56	0.53	0.49
**R_705_/(R_717_+R_491_)**	0.67	0.71	0.65	0.54	0.51	0.59	0.42	0.46	0.30	0.31
**TBDR**	0.67	0.63	0.72	0.59	0.63	0.49	0.57	0.50	0.51	0.49
**MCARI/MTVI2**	0.64	0.63	0.41	0.54	0.47	0.47	0.51	0.47	0.42	0.43
**TCARI/OSAVI**	0.48	0.51	0.32	0.43	0.39	0.37	0.41	0.38	0.27	0.24

The results were accurate to 0.01.

**Table 4:** Coefficients of determination (R^2^) for the relationship between the vegetation indices and the leaf nitrogen concentration in different planting densities.

	LNC_Canopy_			LNC_L1_			LNC_L2_			LNC_L3_			LNC_L4_		
	D1	D2	D3	D1	D2	D3	D1	D2	D3	D1	D2	D3	D1	D2	D3
**RVI**	0.77	0.63	0.69	0.65	0.55	0.62	0.69	0.51	0.54	0.61	0.55	0.58	0.62	0.46	0.49
**RDVI**	0.70	0.60	0.55	0.73	0.63	0.68	0.66	0.58	0.57	0.47	0.45	0.38	0.36	0.24	0.25
**NDVI**	0.78	0.66	0.70	0.68	0.58	0.65	0.69	0.54	0.53	0.60	0.55	0.56	0.58	0.45	0.48
**CI**	0.32	0.04	0.24	0.49	0.11	0.38	0.32	0.06	0.29	0.16	0.02	0.12	0.19	0.00	0.08
**OSAVI**	0.78	0.67	0.70	0.69	0.59	0.67	0.71	0.55	0.55	0.61	0.57	0.57	0.58	0.45	0.47
**NDRE**	0.54	0.38	0.37	0.61	0.44	0.51	0.55	0.41	0.45	0.34	0.29	0.25	0.31	0.19	0.20
**SIPI**	0.28	0.03	0.22	0.45	0.10	0.34	0.28	0.05	0.25	0.13	0.01	0.10	0.16	0.01	0.05
**MTVI2**	0.39	0.33	0.26	0.18	0.16	0.12	0.29	0.22	0.13	0.36	0.36	0.34	0.06	0.28	0.17
**MCARI**	0.41	0.40	0.35	0.24	0.24	0.24	0.36	0.30	0.22	0.35	0.42	0.39	0.05	0.29	0.15
**TCARI**	0.78	0.66	0.69	0.67	0.56	0.63	0.68	0.53	0.52	0.62	0.56	0.57	0.60	0.46	0.50
**NDDA**	0.76	0.68	0.65	0.63	0.56	0.61	0.63	0.51	0.45	0.53	0.53	0.48	0.35	0.32	0.33
**R_705_/(R_717_+R_491_)**	0.76	0.62	0.61	0.67	0.48	0.53	0.69	0.49	0.45	0.57	0.57	0.54	0.52	0.50	0.39
**TBDR**	0.67	0.56	0.54	0.47	0.38	0.41	0.55	0.41	0.34	0.55	0.54	0.53	0.43	0.45	0.40
**MCARI/MTVI2**	0.56	0.52	0.49	0.49	0.35	0.40	0.48	0.39	0.34	0.44	0.51	0.48	0.16	0.36	0.24
**TCARI/OSAVI**	0.77	0.63	0.69	0.65	0.55	0.62	0.69	0.51	0.54	0.61	0.55	0.58	0.62	0.46	0.49

The result were accurate to 0.01.

There were some differences in VIs for different rice cultivars. For V1, the optimal vegetation index for estimating the LNC_Canopy_ was R_705_/(R_717_+R_491_) (R^2^ = 0.704); NDRE was the best vegetation index for LNC_L1_ estimation with the highest value (0.646) of R^2^; R_705_/(R_717_+R_491_) also performed best in the estimation of LNC_L2_ (R^2^ = 0.631); and the optimal VIs for estimating LNC_L3_ and LNC_L4_ were MCARI/MTVI2 (R^2^ = 0.563) and NDDA (R^2^ = 0.627), respectively. For V2, NDRE performed best in estimating LNC_Canopy_ and LNC_L1_, with R^2^ values of 0.728 and 0.723, respectively; the optimal vegetation index for estimating LNC_L2_ was NDVI (R^2^ = 0.574); RVI performed best in estimating LNC_L3_ and LNC_L4_, with R^2^ values of 0.541 and 0.501, respectively. Due to the high accuracy and stability between different treatments, NDDA and NDRE were selected to estimate LNC_Canopy_ (**Figure 10A**) and LNC_L1_ (**Figure 10B**) and were used in the construction of methods 1 and 2 in Section 2.5.

**Figure 9:**
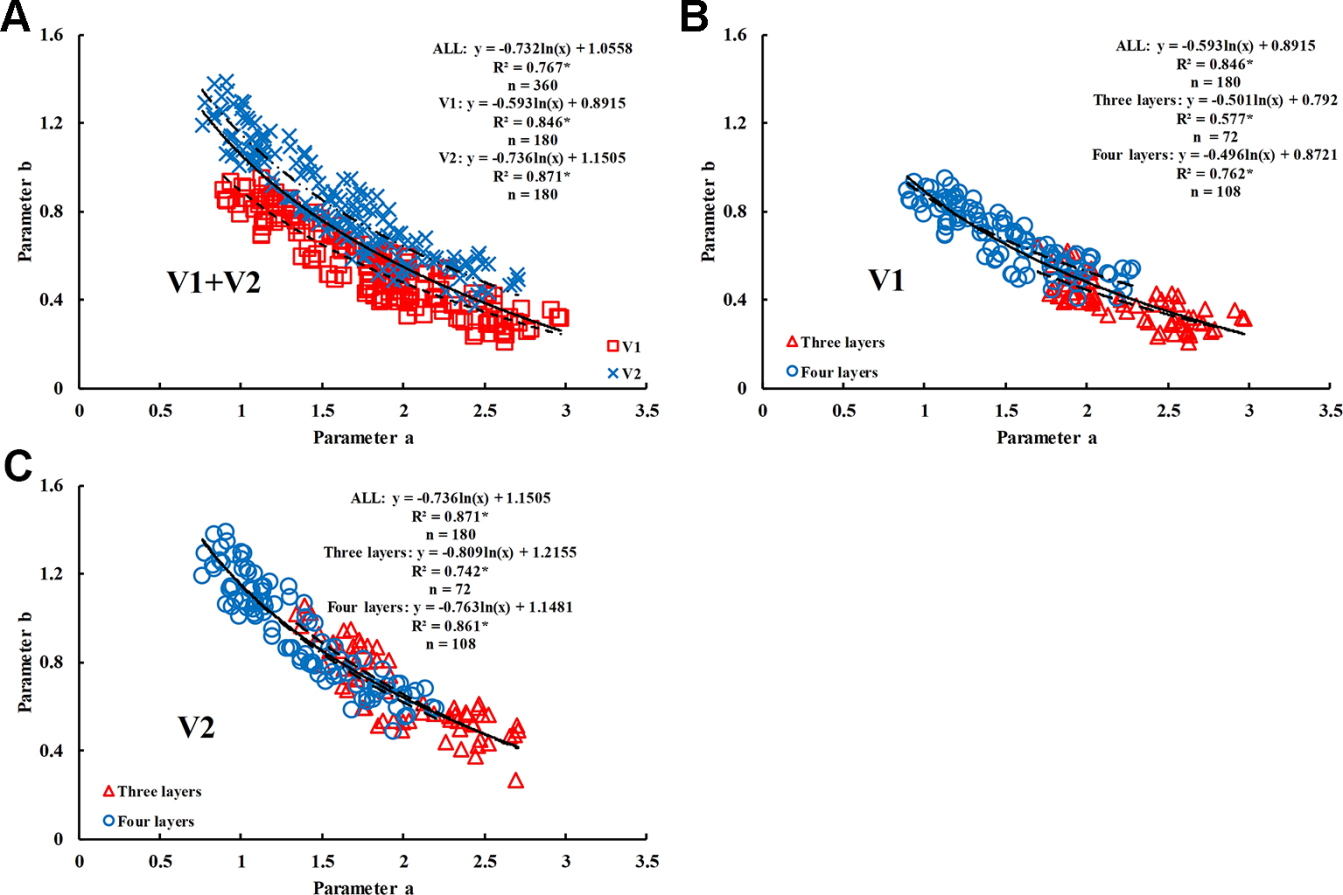
Relationship between parameter ‘a' and ‘b' in different rice varieties. **(A)** V1+V2, **(B)** V1, and **(C)** V2. * means accurate to 0.001.

**Figure 10:**
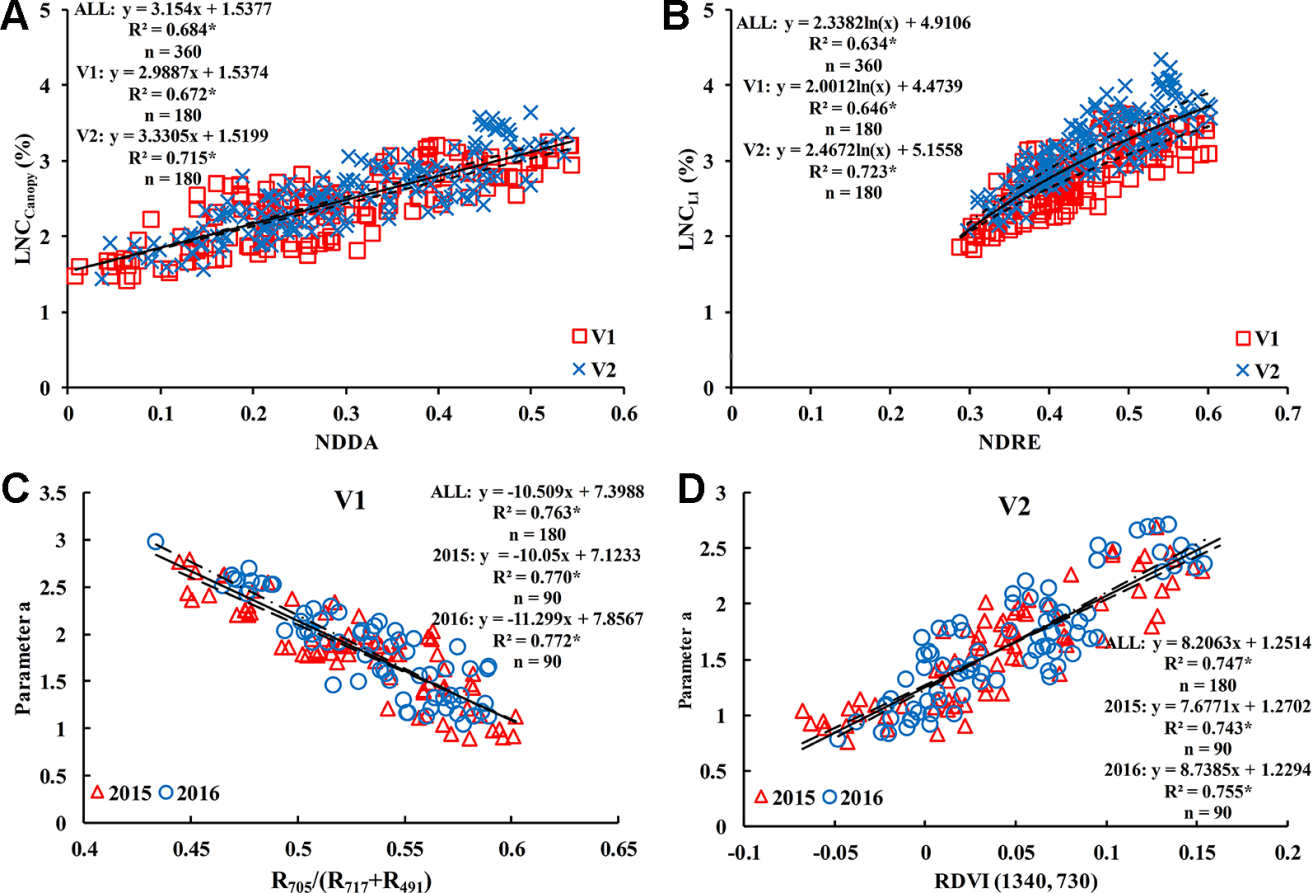
Optimum vegetation indices for estimating LNC_Canopy_ **(A)**, LNC_L1_ **(B)**, and parameter “a” [**(C)** is V1 and **(D)** is V2]. * means accurate to 0.001.

Due to the good correlation between parameter “a” and parameter “b” (**Figure 9A–C**), and the difference in the correlation for different rice varieties, four representative two-band VIs (DI, SR, NDVI, and RDVI) were selected to conduct the 350 nm–1500 nm sensitivity analyses of parameter “a.” The results showed that R_705_/(R_717_+R_491_) and RDVI (1340, 730) were suitable for the estimation of parameter “a” in V1 (R^2^ = 0.763) (**Figure 10C**) and V2 (R^2^ = 0.747) (**Figure 10D**), respectively. Based on these results, parameter “b” could be obtained by the relationship between parameter “a” and “b”.

### LNC_Li_ Estimation of Rice Canopy

The following three methods were compared for estimating the LNC_Li_ within the rice canopy:

(1) estimate the LNC_Canopy_ by the NDDA (**Figure 10A**) and then estimate the LNC_Li_ based on the relationship between the LNC_Li_ and LNC_Canopy_ (**Figure 5**):

(6) $$LNC_{Canopy}=3.154\times NDDA+1.5377$$

(7) $$LNC_{L1}=0.9657\times LNC_{Canopy}+0.5841$$

(8) $$LNC_{L2}=0.862\times LNC_{Canopy}+0.3998$$

(9) $$LNC_{L3}=0.8134\times LNC_{Canopy}+0.1075$$

(10) $$LNC_{L4}=0.8924\times LNC_{Canopy}-0.4005$$

(2) estimate the LNC_L1_ by the NDRE (**Figure 10B**), input the results into the LNC vertical distribution model to obtain the model parameters, and then estimate the LNC_Li_ by the LNC vertical distribution model:

(11) $$LNC_{L1}=2.3382\times ln\left(NDRE\right)+4.9106$$

(12) $$LNC_{L1}=a\times exp\left(b\times H_{r1}\right)$$

(13) b=−0.732×ln(a)+1.0558

(5) $$LNC_{Li}=aexp\left(bH_{ri}\right)$$

(3) directly estimate the model parameters using R_705_/(R_717_+R_491_) (**Figure 10C**) and RDVI (1340, 730) (**Figure 10D**) and then estimate the LNC_Li_ by the LNC vertical distribution model:

(14) $$a=\{\begin{array}{l} -10.509\times \left(\frac{R_{705}}{R_{17}+R_{491}}\right)+7.3988,\;V1\\ 8.2063\times RDVI+1.2514\;,\;V2 \end{array}$$

(13) b=−0.732×ln(a)+1.0558

(5) $$LNC_{Li}=aexp\left(bH_{ri}\right)$$

**Figure 11:**
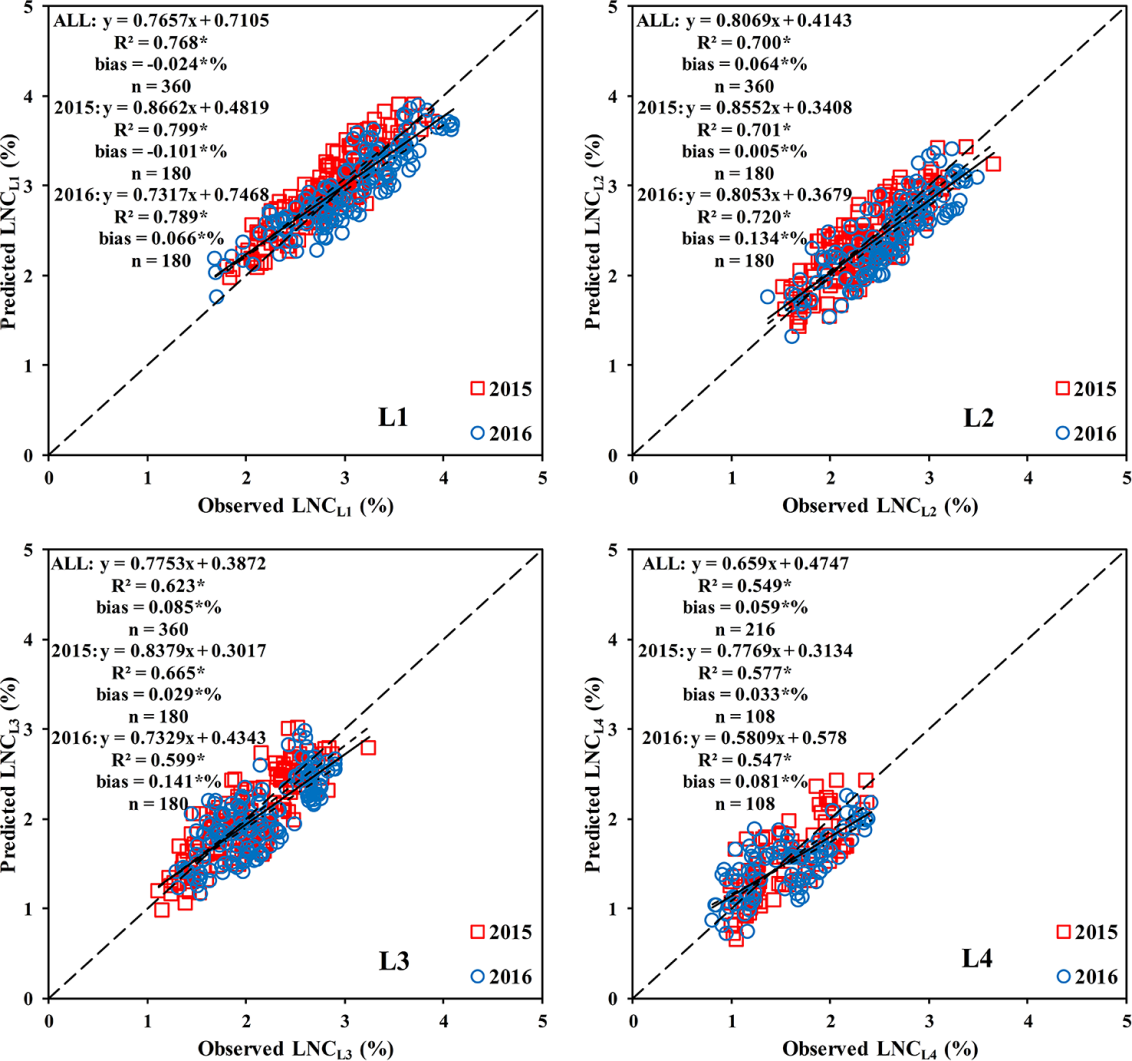
Comparison between the observed and predicted LNC in different leaf layers based on method 2. * means accurate to 0.001.

The results (**Figure 11** and **Supplementary Figures 3** and **4**) showed that all three methods could estimate the LNC_Li_ within the rice canopy; however, method 1 significantly underestimated the LNC_L1_. The methods (2 and 3) that combined the LNC vertical distribution model performed better in estimating the LNC_Li_. Method 2 was the optimal method for estimating the LNC_L1_, LNC_L2_, LNC_L3_, and LNC_L4_, which had R^2^ values of 0.768, 0.700, 0.623, and 0.549, respectively.

## Discussion

In this study, the temporal and spatial distribution characteristics of the LNC in the rice canopy at different growth stages were investigated. Our study results showed that the LNC distribution followed a declining trend from the top layer to the bottom layer in rice canopy (**Figure 3**), which is consistent with the findings in wheat (Li et al., 2015; Zhao et al., 2017), winter oilseed rape (Li et al., 2018), and maize (Ye et al., 2018). Light conditions are the main limiting factors for vegetation photosynthesis, with more N allocated to leaves receiving higher light intensities (Wyka et al., 2012); thus, the top of the canopy, which has the best light conditions in the rice canopy, is assigned more. N. Chen et al. (2014) reported the light use efficiency (LUE) is lower in the bottom leaf layers than the top fresh leaf layers because the lower leaves of the canopy are more sensitive to nutrient stress and senescence. Therefore, the LNC and LNC_R_ were the lowest in the bottom leaf layer of the rice canopy (**Figures 3** and **4**). In the high N treatments (N2), L2 and L3 had higher LAI and more sunlit leaves compared with the low N treatment, which led to a higher LNC_L1_ and a lower LNC_R1_ in the high N treatments than those in the low N treatments (**Figure 4**). In the low planting density (D3), the plant spacing is larger than the high planting density, and more light can reach the lower leaf layer of rice canopy. Therefore, compared with high density treatments, low density treatments distributed more N in the lower leaf layer of the rice canopy. These may explain why the relative variation rates of LNC decreased gradually with the decreased planting densities for the bottom leaf layer of the canopy (**Figure 7**). V1 (with erect leaf) and V2 (with spread leaf) performed differently, and the leaves of V2 were mainly concentrated in the upper layer of the canopy, causing less light in the lower part of the canopy. Therefore, for V2, the vertical distribution trend of N was steeper and the low density treatment increased the LNC of lower leaf layers more significantly.

Due to the transferability of N (Wang et al., 2005), the N of the bottom leaf layer is transferred to the top leaf layer when the N supply is limited (Wang et al., 2006). Therefore, the upper layers were less affected by the N rates, and N stress was more obvious in the lower layers, which is similar to the findings of Huang et al. (2014). Kong et al. (2017) also showed that accurate estimations of the physiological and biochemical parameters of the bottom leaves can facilitate the early detection of subtle changes in the field. Studies also showed that the nitrogen stress index (NSI) and the nitrogen spectral stress index (NSSI) of the bottom two leaves could indicate the N stress in maize (Lu et al., 2018). D'Odorico et al. (2019) reported that bottom leaves showed higher photosynthetic efficiency at low light levels and were the main contributor to total canopy photosynthesis until the start of senescence. The LNC of the bottom leaf layer was significantly lower than the LNC_Canopy_, and the LNC_Canopy_ was mainly affected by the LNC of the upper and middle leaf layers (**Figures 5** and **6**). Therefore, the LNC_Canopy_ could not rapidly show whether crops were under N stress, which indicates the importance of accurately estimating the LNC of each layer within the canopy.

The LNC vertical distribution model proposed in this study could simulate the vertical distribution of the LNC within the rice canopy (**Table 1**). The difference in light distribution caused by differences in the canopy structure (e.g., LAI and leaf angle) is the main reason for the non-uniform N distribution (Archontoulis et al., 2011; Hikosaka et al., 2016; Ye et al., 2018), and the relationship between the N distribution and light distribution differs at different growth stages (Dreccer et al., 2000). Therefore, the parameters of our model were susceptible to the growth period and rice variety (**Table 1**).

Previous studies seldom considered the vertical distribution of N within the canopy (Li et al., 2016). Our studies showed that the estimation accuracy of LNC_Li_ based on VIs decreased as the depth of the canopy increased, and the upper and middle layers were the effective leaf layers for LNC predictions using VIs. This finding is consistent with the results of Li et al. (2018) in the winter oilseed rape. Luo et al. (2016) reported that the spectral reflectance of the crop canopy is mainly affected by the upper layer in the visible and near-infrared bands, which may explain why the VIs (e.g., SIPI) based on red and near-infrared bands are less effective at estimating the LNC of the bottom leaf layers. VIs that include the red edge band showed a close correlation with the LNC of different leaf layers, which is consistent with the findings of Zhao et al. (2017) in winter wheat. The RVI (780, 740), which was proposed to estimate N absorption in different maize leaf layers (Winterhalter et al., 2012), performed well in estimating the LNC of the lower leaf layer in the rice canopy; however, it did not perform stably between different rice varieties. Canopy structure, physiological and biochemical characteristics of target objects, and background affect the estimation accuracy of VIs by affecting canopy reflectance (Zarco-Tejada et al., 2001). The physiological and biochemical characteristics of crops and the size of soil and water background are mainly affected by N rates and planting density. The higher the proportion of vegetation signal in canopy reflectance, the higher the accuracy of VIs in estimating physiological and biochemical status of vegetation. Therefore, VIs showed a high accuracy in LNC_Canopy_ and LNC_Li_ estimation under the high planting density (**Table 4**). Under the same N rates, the N obtained by a single plant increased with the decreased planting density. At the same time, the proportion of background and the size of individual vegetation canopy were constantly changing, which was a complex process. As a result, the VIs that are sensitive to canopy structure had different change modes compared with the VIs that are sensitive to leaf color. The canopy reflectance is bidirectional, and different canopy structures often lead to different variations of canopy reflectance (Li et al., 2015; Zhao et al., 2017). Different rice varieties have various plant types and leaf angles, which affected the canopy structure and also changed the N vertical distribution gradient. Therefore, VIs have significant differences between the two rice cultivars (**Table 2**).

The accuracy of estimating the LNC_Li_ by VIs is limited due to the inconsistency of the optimal VIs and optimal band range among different leaf layers within the canopy, and different remote sensing models must be developed for different layers, although such models are difficult to apply in practice. Therefore, three methods for estimating the LNC_Li_ were constructed and compared in this paper (Section 2.5). Method 1 had a significant underestimation of the LNC_L1_ and performed poorer than methods 2 and 3, which combine the LNC vertical distribution model for estimating the LNC_Li_. Method 3 is subject to the empirical relationship between parameter “a” and the VIs, and it is greatly influenced by external factors (e.g., varieties). Compared with methods 1 and 2, the universality of method 3 must be validated using additional data. Method 2 only needs to accurately estimate the LNC of any leaf layer within the canopy, and the results are then combined with the LNC vertical distribution model, which is a high-precision method with few input parameters and easy calculation, and it allows the LNC at any height of the canopy to be obtained with higher precision. Therefore, it is the optimal choice for obtaining the vertical distribution of the LNC within the rice canopy because it estimates the LNC_L1_ and combines it with the LNC vertical distribution model.

## Conclusion

In the present study, we investigated the vertical distribution characteristics of the LNC within the rice canopy and the relationships of the LNC_Canopy_ with the LNC_Li_ and LNC_ALi_ and proposed an LNC vertical distribution model based on the relative canopy height. The performance of multiple VIs in the LNC_Li_ and model parameter “a” estimation was evaluated at different growth stages. We also compared the three methods for estimating the LNC in different leaf layers within the rice canopy. The results demonstrated that the LNC presented a decreasing trend from top to bottom within the rice canopy, the LNC in the bottom of rice canopy was more susceptible to different N rates, and the LNC_Li_ could be better simulated in different varieties, treatments, and growth stages by using the LNC vertical distribution model. The NDRE could accurately estimate the LNC_L1_, and in combination with the LNC vertical distribution model, the parameters of the model could be accurately obtained, which promotes more accurate estimations of the LNC_Li_ during the whole growth period. The results provide technical support for predicting the spatial and temporal LNC distribution of rice.

## Data Availability Statement

The datasets generated for this study are available on request to the corresponding author.

## Author Contributions

JH and YT conceived and designed the experiments. XZ, WG, and YP performed the experiments. JH analyzed the data. JH and YT wrote the original manuscript. YT, XY, TC, YZ, and WC reviewed and edited the original manuscript.

## Funding

This work was supported by the National Natural Science Foundation of China (31971784), the National Key R&D Program (2018YFD0300805), and the Priority Academic Program Development of Jiangsu Higher Education Institutions (PAPD).

## Conflict of Interest

The authors declare that the research was conducted in the absence of any commercial or financial relationships that could be construed as a potential conflict of interest.

## Abbreviations

Ali, The ith accumulative leaf layer; CI_red_ _edge_, Chlorophyll index red-edge; DW, The dry weight; Hr, The relative canopy height; Li, The ith leaf layer; LAI, Leaf area index; LDW, Leaf dry weight; LNC, Leaf nitrogen concentration; LNC_Canopy_, LNC of the canopy; LNC_Li_, LNC of the ith leaf layer; LNC_R_, LNC ratio; LNC_Ri_, LNC ratio of the ith leaf layer; LND, Leaf nitrogen density; LUE, Light use efficiency; MCARI, Modified chlorophyll absorption in reflectance index; MTVI2, Modified triangular vegetation index 2; N, Nitrogen; N_L_, Nitrogen content per unit leaf area; NDDA, Normalized difference index of the double-peak areas; NDRE, Normalized difference red-edge; NDVI, Normalized difference vegetation index; NSI, Nitrogen stress index; NSSI, Nitrogen spectral stress index; OSAVI, Optimized soil adjusted vegetation index; R^2^, Determination coefficient; RDVI, Renormalized difference vegetation index; RMSE, Root mean square error; RVI, Ratio vegetation index; SIPI, Structure intensive pigment index; TBDR, Three-band difference ratio; TCARI, Transformed chlorophyll absorption ratio index; Vis, Vegetation indices; Z_D_, The depth from top of canopy.
