# Supplementary material for: Estimation of Vertical Leaf Nitrogen Distribution Within a Rice Canopy Based on Hyperspectral Data
Source: Front Plant Sci. 2020 Feb 13;10:1802. doi: 10.3389/fpls.2019.01802 (PMC7031418; doi:10.3389/fpls.2019.01802)
Supplement: Supplementary file 1 [file DataSheet_1.docx]

Supplementary Material

# Supplementary Figures and Tables

## Supplementary Figures


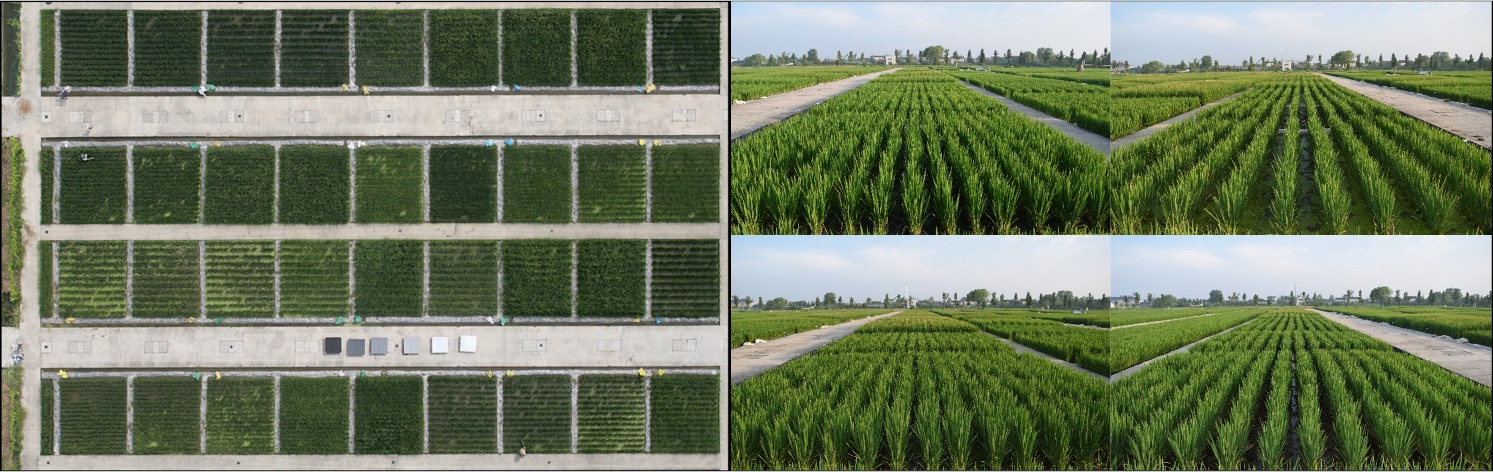


**Supplementary Figure 1.** Views of field plots.


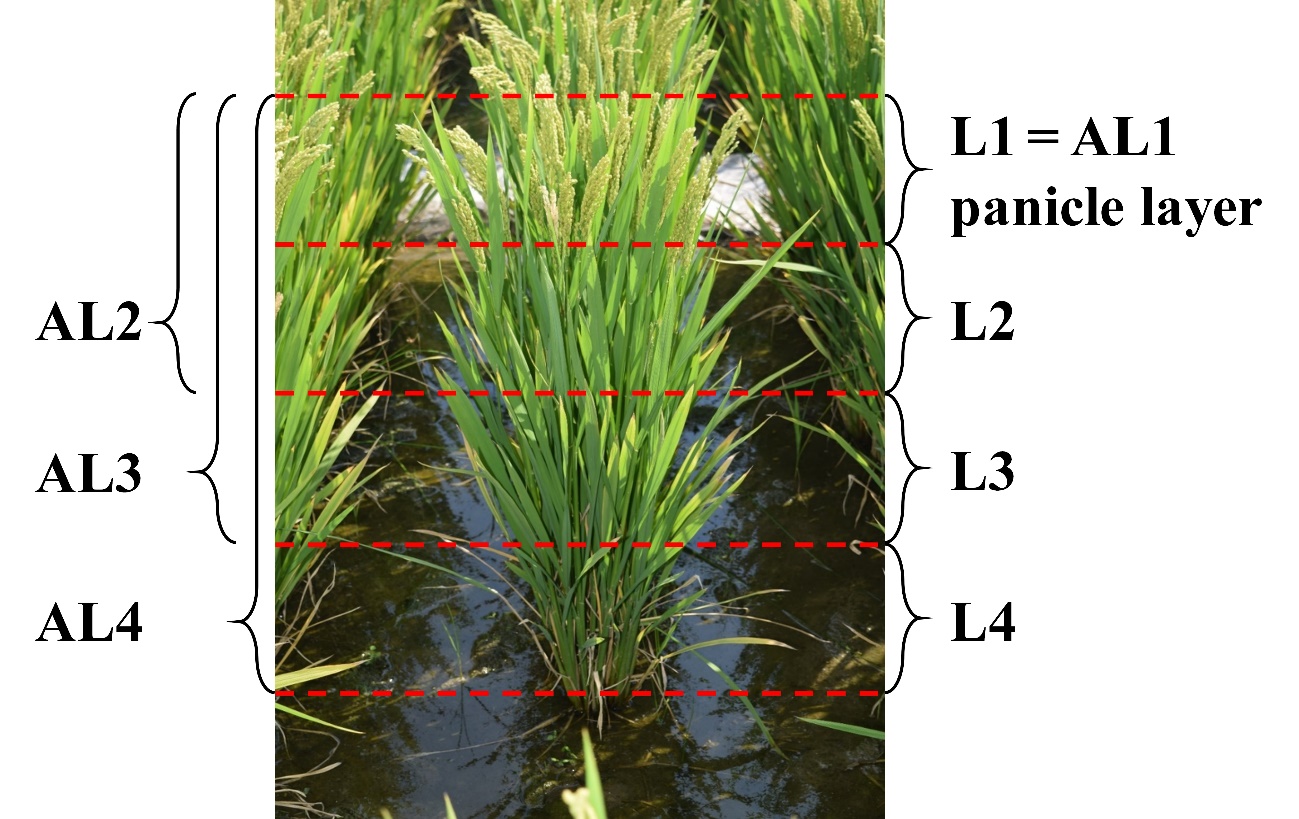


**Supplementary Figure 2.** Conceptual diagram of vertical layer division within the rice canopy (He et al., 2019).

**
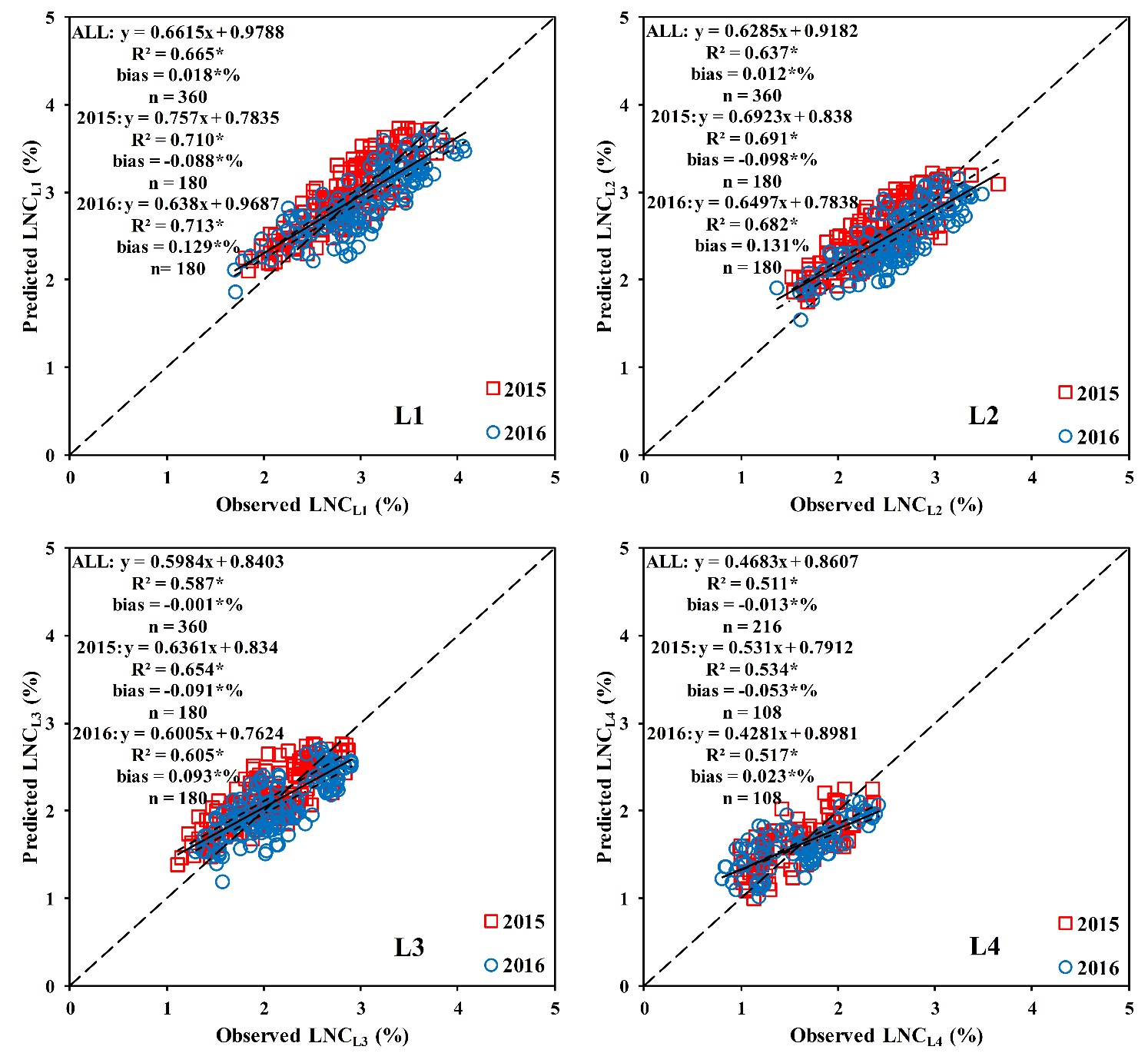
**

**Supplementary Figure 3.** Comparison between the observed and predicted LNC in different leaf layers based on method 1.

**
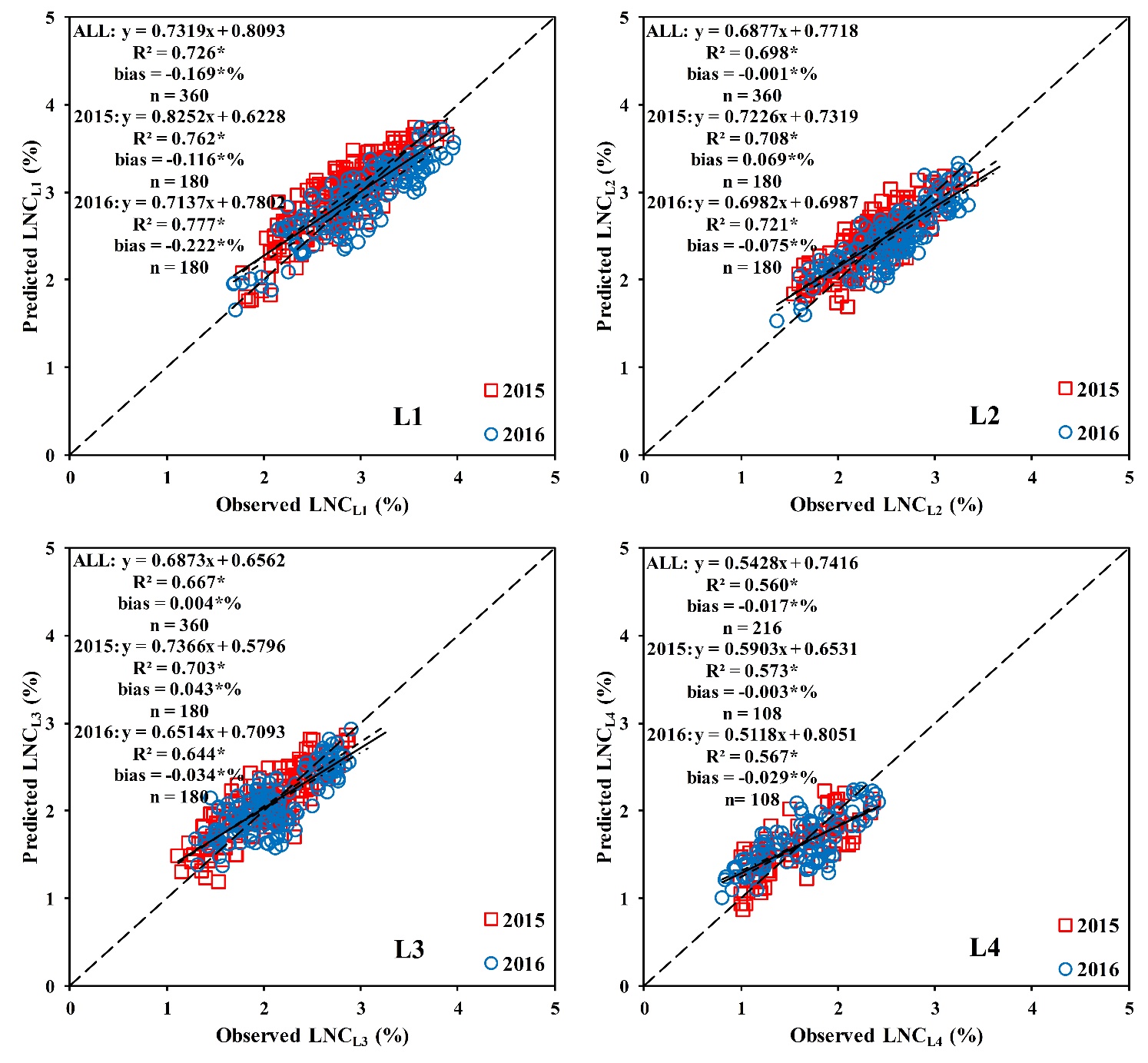
**

**Supplementary Figure 4.** Comparison between the observed and predicted LNC in different leaf layers based on method 3.

## Supplementary Tables

**Supplementary Table 1.** Detailed information on the field treatments adopted in the present study.

|  | **Cultivar** | **Nitrogen fertilization rate (kg N ha^-1^)** | **Planting density (plants m^-2^)** | **Sampling & spectral measurement data (DAT^a^)** |
| --- | --- | --- | --- | --- |
| **Exp.1 (2015)** | V1: Wuyunjing 24  (WYJ 24)  V2: Eryou 728  (EY 728) | N1: 100  N2: 300 | D1: 22.22  D2: 16.66  D3: 13.33 | 58, 71, 86, 95, 101 |
| **Exp.2 (2016)** | V1: Wuyunjing 24  (WYJ 24)  V2: Eryou 728  (EY 728) | N1: 100  N2: 300 | D1: 22.22  D2: 16.66  D3: 13.33 | 59, 73, 87, 96, 103 |

^a^ DAT: Days after transplanting

**Supplementary Table 2.** Spectral vegetation indices used in this study.

|  | **Vegetation index** | **Formula** | **Reference** |
| --- | --- | --- | --- |
| Double-band VIs | RVI | $R_{780}/R_{740}$ | Winterhalter et al., 2012 |
|  | RDVI | $(R_{800}-R_{670})/\sqrt{(R_{800}+R_{670})}$ | Roujean & Breon, 1995 |
|  | NDVI | $(R_{800}-R_{670})/(R_{800}+R_{670})$ | Rouse et al., 1974 |
|  | CI_red edge_ | $(R_{750}/R_{720})-1$ | Gitelson et al., 2005 |
|  | OSAVI | $(1+0.16)(R_{800}-R_{670})/(R_{800}+R_{670}+0.16)$ | Rondeaux et al., 1996 |
|  | NDRE | $(R_{790}-R_{720})/(R_{790}+R_{720})$ | Barnes, 2000 |
| Multi-band VIs | SIPI | $(R_{800}-R_{445})/(R_{800}+R_{680})$ | Penuelas et al., 1995 |
|  | MTVI2 | $\frac{1.5[1.2\left( R_{800}-R_{550} \right)-2.5(R_{670}-R_{550})]}{\sqrt{{(2R_{800}+1)}^{2}-\left( 6R_{800}-5\sqrt{R_{670}} \right)-0.5}}$ | Haboudane et al., 2004 |
|  | MCARI | $(R_{700}-R_{670}-0.2(R_{700}-R_{550}))(R_{700}/R_{670})$ | Daughtry et al., 2000 |
|  | TCARI | $3(\left( R_{700}-R_{670} \right)-0.2\left( R_{700}-R_{550} \right)\left( \frac{R_{700}}{R_{670}} \right))$ | Haboudane et al., 2002 |
|  | NDDA | $(R_{680}+R_{756}-2R_{718})/(R_{756}-R_{680})$ | Feng et al., 2014 |
|  | R_705_/(R_717_+R_491_) | $R_{705}/(R_{717}+R_{491})$ | Tian et al., 2011 |
|  | TBDR | $R_{755}/(R_{513}-R_{508})$ | Wang et al., 2019 |
| Combined Index | MCARI/MTVI2 | ${MCARI}/{MTVI2}$ | Eitel et al., 2007 |
|  | TCARI/OSAVI | ${TCARI}/{OSAVI}$ | Haboudane et al., 2002 |

**Supplementary Table 3.** Basic statistics of the canopy height (cm) for 2015 and 2016.

| **Cultivar** | **Sampling Time^*^** | **Mean** | **SD** | **Min.** | **Max.** |
| --- | --- | --- | --- | --- | --- |
| V1 | 1^st^ | 66.14 | 4.62 | 56 | 75 |
|  | 2^nd^ | 75.94 | 4.59 | 69 | 86 |
|  | 3^rd^ | 86.53 | 7.06 | 76 | 104 |
|  | 4^th^ | 84.08 | 8.04 | 72 | 99 |
|  | 5^th^ | 83.17 | 7.30 | 70 | 97 |
| V2 | 1^st^ | 87.69 | 9.22 | 68 | 101 |
|  | 2^nd^ | 96.33 | 8.24 | 80 | 109 |
|  | 3^rd^ | 112.50 | 6.56 | 100 | 125 |
|  | 4^th^ | 111.00 | 8.20 | 94 | 122 |
|  | 5^th^ | 110.47 | 8.71 | 93 | 123 |

*The five sampling times in 2015 and 2016 were 58, 71, 86, 95, 101 DAT and 59, 73, 87, 96, 103DAT
